# Posttranslational forms of beta 2-glycoprotein I in the pathogenesis of the antiphospholipid syndrome

**DOI:** 10.1186/s12959-016-0115-z

**Published:** 2016-10-04

**Authors:** Fatima El-Assaad, Steven A. Krilis, Bill Giannakopoulos

**Affiliations:** 1 Department of Infectious Diseases, Immunology, and Sexual Health, St. George Hospital, Level 1, 2 South Street, Kogarah, NSW 2217 Australia; 2 St. George and Sutherland Clinical School, Faculty of Medicine, University of New South Wales Australia, Research and Education Centre, Level 3, 4 - 10 South St, Kogarah, NSW 2217 Australia

**Keywords:** Antiphospholipid syndrome, Beta-2 Glycoprotein-1, Oxidative modification

## Abstract

The antiphospholipid syndrome (APS) is an autoimmune disease characterised by a procoagulant state that predisposes to recurrent thrombosis and miscarriages. Two major discoveries have advanced our understanding of the underlying complex pathogenesis of the APS. The first was the discovery that beta-2 glycoprotein-1 (β2GPI) is the major auto antigen in APS. The second was the discovery in more recent years that β2GPI contains allosteric disulphide bonds susceptible to posttranslational modification that may be involved in the development of autoantibodies in APS. The main allosteric disulphide bond in the fifth domain of β2GPI can exist in two redox states: free thiol or oxidised. It is the conformational transformation of β2GPI from its free thiol form to its more immunogenic oxidised form that exposes neo-epitopes on the first and fifth domains. The purpose of this review is to highlight the recent findings on the posttranslational forms of β2GPI in the pathogenesis of APS. We suggest that novel assays quantitating the different redox forms of β2GPI in plasma or serum may be used to supplement existing clinical and laboratory assays to more accurately stratify risk of thrombosis or miscarriage in APS patients.

## Background

Antiphospholipid syndrome (APS) is an autoimmune disease and a major cause of acquired thrombophilia. It is characterised by a hypercoaguable vascular compartment due to the persistent presence of antiphospholipid antibodies (aPL), detected by either one of the following assays: in vitro coagulation assay lupus anticoagulant (LA), and/or solid phase assays anticardiolipin (aCL) and/or anti-beta 2-glycoprotein I (anti-β2GPI) [1, 2].

APS has two main manifestations: thrombotic or obstetric [3]. The hallmark feature of thrombotic APS is arterial or venous thrombosis, most commonly stroke and/or lower limb venous thromboembolism. Obstetric APS presents as recurrent first trimester miscarriages, and second trimester foetal death and obstetric complications such as early onset severe preeclampsia. APS can be associated with other autoimmune diseases such as systemic lupus erythematosus (SLE). In rare cases this syndrome can develop into an often fatal manifestation known as catastrophic APS, characterized by thrombotic microangiopathy in multiple concurrent vascular beds.

## Review

### Diagnosis and treatment of APS

Renewed efforts to understand the pathogenesis and streamline the diagnosis and treatment regimen has been the focus of clinicians and researchers in the field globally [4]. Current consensus is to diagnose APS using a combination of laboratory and clinical criteria [2, 5]. APS classification is made when two serological tests 12 weeks apart return positive for persistent levels of lupus anticoagulant (LA), or high titres anticardiolipin (aCL) and/or anti-β2GPI antibodies in combination with a history of a thromboembolic event or miscarriages [6].

There is no single treatment for APS. The gold standard therapeutic management comprises of antiplatelet and anticoagulant agents, although no consensus exists on the finite duration and intensity regimen as reviewed in [5, 7]. Corticosteroids, plasma exchanges are also considered in patients presenting with catastrophic APS [8].

### Beta 2-glycoprotein I is the major autoantigen in APS

β2GPI, also known as apolipoprotein H, is the main autoantigen in APS [9]. It mediates the binding between aPL and phospholipids. It was previously thought that aPL bound directly to phospholipids resulting in the inaccurate naming of this syndrome [9]. aPL only recognise β2GPI when it is bound to phospholipids as cryptic epitopes become exposed following binding.

### Beta 2-glycoprotein I biology

β2GPI is important in the pathogenesis of the APS, but its precise physiological function in vivo is still being delineated [10, 11]. There is evidence to show that it regulates complement [12], inhibits procoagulant factors such as factor XI/XIa and thrombin [13–15]. It inhibits angiogenesis [16, 17] and interacts with components of the atherosclerotic plaque such as apolipoprotein(a) [18]. It interacts with the platelet receptor glycoprotein Ib-IX-V [19] and with von Willebrand Factor (vWF) [20]. It also interacts with endothelial cells [21, 22] and apoptotic cells [23]. β2GPI is also involved in the innate immune response and plasma levels drop during the acute phase of infective inflammation [24]. It also plays a role in early embryonic development and is important for implantation success in mice [25]. β2GPI can interact with apolipoprotein E receptor 2 (APOER2), a member of the low-density lipoprotein (LDL) receptor family as well as annexin V and II on cell membranes of platelets, endothelial cells and monocytes [26–28].

β2GPI is synthesised in the liver and can localise on the endothelium of uterine vessels and the site of implantation in pregnant mice [29]. There is some evidence for β2GPI expression in the intestines of rats and mice and low expression in rat kidney, heart, brain, spleen, stomach and prostate [30, 31]. However, only following priming with lipopolysaccharide (LPS) does β2GPI localise on cerebral, cardiac and intestinal endothelial cells [29]. It can be detected at the site of injury within the retina of patients with age-related macular degeneration (AMD) [32] and the myocardium of mice subjected to ischemia reperfusion injury (IRI) [33].

The absence of β2GPI modulates the autoimmune phenotype in SLE prone mice in which toll like receptor 7 (TLR7) plays a major role [34]. In a mouse model of systemic lupus, deletion of β2GPI increased the levels of autoantibodies, inflammatory cytokines and the presence of lymphadenopathy and splenomegaly [34]. In a mouse model of cardiac IRI, damage is reduced by treatment with the fifth domain of β2GPI [33]. Treatment with the fifth domain of β2GPI also reduced neutrophil and monocyte infiltration in the area at risk following reperfusion [33]. Domain V offers potential as a therapy in cardiac IRI by acting as a universal inhibitor of the initial inflammatory cascade by blocking the binding of pathogenic natural IgM antibodies to cardiac neoepitopes exposed during reperfusion [33].

### Structural conformations of Beta 2-glycoprotein I

Human β2GPI is highly conserved and has high structural homology between species [35]. It consists of five domains, domain I at the N-terminus through to domain V at the C-terminus. These domains are also known as short consensus repeats (SCR), sushi domains or complement control proteins. The first four domains are made up of 60 amino acid residues with the fifth domain having an extra 26 amino acid loop. β2GPI has 11 disulphide bonds in total, two disulphide bonds per domain except for the fifth domain that contains an extra disulphide bond.

The fifth domain is exceptionally important as the extra disulphide bridge within it participates in the preferential binding of negatively charged phospholipids on activated cell membranes [36, 37]. The unique structural feature of domain V is the aberrant fold made of a six-residue insertion and a 19-residue extension C‐terminally cross‐linked by an extra disulfide bond between Cys288-Cys326 [36, 38]. There are two main reasons for the unique phospholipid membrane binding specificity of the fifth domain. Firstly, the fifth domain has a positively charged patch. Secondly, it has a flexible partially hydrophobic insertion loop that inserts into the lipid layer and uses Trp316 to anchor into the membrane. This insertion loop is highly conserved across the entire animal kingdom suggesting its physiological importance [35].

### Posttranslational modification of Beta 2-glycoprotein I

The extra disulphide bond Cys288-Cys326 in the C terminal domain is allosteric and mediates a functional change of the protein following posttranslational modification [20, 39]. β2GPI has naturally occurring free thiols in vivo and the catalytic disulphide bonds found in oxidoreducatses can control the redox state of this bond [40, 41]. This allosteric disulphide bond influences the structure and function of β2GPI.

β2GPI is a thiol oxidoreductase substrate and its cysteine residue (Cys288–Cys326) within the fifth domain is able to form free thiols following incubation with oxidoreducatase enzymes thioredoxin-1 (TRX-1) and protein disulphide isomerase (PDI) [40]. Thiol oxidoreductases are important in platelet and endothelial cell function [40, 42].

It has been proposed that β2GPI has a major role in the pathogenesis of APS due to its susceptibility to posttranslational oxidative modification by reactive oxygen species and the subsequent exposure of neoantigens [3]. β2GPI protects endothelial cells from oxidative stress-induced cell death [41] and promotes platelet vWF interactions in vitro [20]. Under oxidative stress, free thiol β2GPI undergoes posttranslational modification through oxidation and/or nitrolysation of the redox sensitive cysteine residues [43]. The addition of oxygen or nitrogen oxide (NO) to cysteines can alter the function of β2GPI and increase its immunogenicity. Oxidative modification of β2GPI at supraphysiological concentrations of oxidant has been implicated in promoting helper type 1 T cell responses and inducing the maturation of monocyte-derived dendritic cells [44].

Following posttranslational modification, β2GPI undergoes conformational change that exposes the major B-cell binding site on domain I and the major T-cell binding site on domain V. Thus, the proportion of free thiol β2GPI and oxidized β2GPI is important to the development of anti-β2GPI autoantibodies in APS. Domain 1 and V are required to elicit the generation of pathogenic aPL [3]. Although no direct structural or spectroscopic data demonstrating a conformational change following posttranslational modification of β2GPI have been reported, affinity purified anti-β2GPI autoantibodies from patients with APS bind oxidised-β2GPI at a higher avidity than the free-thiol form [3].

Pathogenic aPL recognise domain I only after the conformational change [45]. Recombinant domain I of β2GPI can inhibit thrombus formation in vivo in a mouse model of anti-β2GPI induced potentiation of mechanical induced thrombosis [46].

### Oxidised Beta 2-glycoprotein I a biomarker for APS?

Quantitation of oxidised APS could be useful as a biomarker of APS. The levels of total β2GPI in serum of APS patients are elevated compared to patients with other autoimmune diseases, thrombosis or healthy controls [39, 47]. These levels are only elevated in patients positive for persistent aPL with a history of thrombosis [39]. The proportion of protective free thiol β2GPI in APS patients is low compared to patients with other autoimmune diseases. Free thiol β2GPI may be protective and the lower levels indicate a higher proportion of oxidised β2GPI [39].

Current classification criteria do not enable stratification of thrombotic risk. Detection of thiol-exchange reactions, the posttranslational modification of β2GPI could supplement current diagnostic tools for APS and accurately predict risk of thrombosis in APS [39]. In vitro assays screening samples to stratify thrombosis risk, could also be modified to assay for free thiols in other diseases where the redox-state of β2GPI is an important player such as age related macular degeneration [32]. Directly quantifying the various redox states of β2GPI is a unique and novel approach for delineating the relationship between autoantigen redox state and disease severity [39].

We have developed two β2GPI enzyme-linked immunosorbent assays (ELISA) one measuring the total level of β2GPI and the other the proportion of free thiol β2GPI in human plasma and serum. Free thiol β2GPI is quantified using a biotinylated probe that is specific for free thiols. Following addition of this probe to plasma or serum, the unincorporated biotinylated probe is removed by an acetone precipitation step and biotinylated proteins are specifically captured on a streptavidin ELISA plate. A monoclonal anti-β2GPI antibody quantifies the amount of β2GPI with biotinylated free thiols that have been captured on the ELISA plate [39]. From these assays we are able to deduce the proportion of β2GPI in an oxidised state [39]. Quantification of β2GPI and its post-translational forms can supplement existing prognostic and diagnostic protocols to better stratify thrombotic risk in APS patients.

## Conclusions

Oxidized β2GPI may be a useful specific prognostic marker for APS and enhance our management of APS patients. In a retrospective multicenter international study we demonstrated that patients with thrombotic APS have higher levels of oxidized β2GPI compared to healthy individuals and patients with other autoimmune conditions [39]. β2GPI is susceptible to cleavage by oxidoreductases that circulate in human plasma and reduce the cysteines to free thiols. These free thiols can be quantified using a novel ELISA assay we have developed and may be useful in the diagnosis of aPL antibody positive patients presenting with no clinical event or in stratifying the risk of a thrombotic event in patients with APS.

## Abbreviations

aCL: Anti-cardiolipin
AMD: Age-related macular degeneration
anti-β2GPI: Anti-beta 2-glycoprotein I
aPL: Antiphospholipid antibodies
ApoER2: Apolipoprotein E receptor 2
APS: Antiphospholipid syndrome
Cys: Cysteine
ELISA: Enzyme-linked immunosorbent assay
IRI: Ischemia reperfusion injury
LA: Lupus anticoagulant
LDL: Low density lipoprotein
MPB: N^a^-(3-maleimidylpropionyl) biocytin
NO: Nitrogen oxide
PDI: Protein Disulfide Isomerase
SCR: Short consensus repeats
SLE: Systemic lupus erythematosus
TLR 7: Toll like receptor 7
TNF: Tumour necrosis factor
TRX-1: Thioredoxin-1
VLDL: Very low density lipoprotein
vWF: von Willebrand Factor
β2GPI: Beta-2 glycoprotein-I
